# Beta-Arrestins in the Treatment of Heart Failure Related to Hypertension: A Comprehensive Review

**DOI:** 10.3390/pharmaceutics13060838

**Published:** 2021-06-05

**Authors:** Ahmed Rakib, Taslima Akter Eva, Saad Ahmed Sami, Saikat Mitra, Iqbal Hossain Nafiz, Ayan Das, Abu Montakim Tareq, Firzan Nainu, Kuldeep Dhama, Talha Bin Emran, Jesus Simal-Gandara

**Affiliations:** 1 Department of Pharmacy, Faculty of Biological Sciences, University of Chittagong, Chittagong 4331, Bangladesh; arakib@uthsc.edu (A.R.); eva663300@gmail.com (T.A.E.); s.a.sami18pharm@gmail.com (S.A.S.); 2 Department of Pharmacy, Faculty of Pharmacy, University of Dhaka, Dhaka 1000, Bangladesh; saikat-2018926336@pharmacy.du.ac.bd; 3 Department of Biochemistry and Molecular Biology, Faculty of Biological Sciences, University of Chittagong, Chittagong 4331, Bangladesh; nafiz.iqbal27cu@gmail.com (I.H.N.); ayan.ae7788@gmail.com (A.D.); 4 Department of Pharmacy, International Islamic University Chittagong, Chittagong 4318, Bangladesh; abu.muntakim@dblab.org; 5 Faculty of Pharmacy, Hasanuddin University, Tamalanrea, Kota Makassar, Sulawesi Selatan 90245, Indonesia; firzannainu@unhas.ac.id; 6 Division of Pathology, ICAR-Indian Veterinary Research Institute, Izatnagar, Bareilly 243122, Uttar Pradesh, India; kdhama@rediffmail.com; 7 Department of Pharmacy, BGC Trust University Bangladesh, Chittagong 4381, Bangladesh; 8 Nutrition and Bromatology Group, Department of Analytical and Food Chemistry, Faculty of Food Science and Technology, University of Vigo–Ourense Campus, E32004 Ourense, Spain

**Keywords:** cardiovascular disease, heart failure, hypertension, β-arrestin, renin–angiotensin–aldosterone system, beta-adrenergic receptors

## Abstract

Heart failure (HF) is a complicated clinical syndrome that is considered an increasingly frequent reason for hospitalization, characterized by a complex therapeutic regimen, reduced quality of life, and high morbidity. Long-standing hypertension ultimately paves the way for HF. Recently, there have been improvements in the treatment of hypertension and overall management not limited to only conventional medications, but several novel pathways and their pharmacological alteration are also conducive to the treatment of hypertension. Beta-arrestin (β-arrestin), a protein responsible for beta-adrenergic receptors’ (β-AR) functioning and trafficking, has recently been discovered as a potential regulator in hypertension. β-arrestin isoforms, namely β-arrestin1 and β-arrestin2, mainly regulate cardiac function. However, there have been some controversies regarding the function of the two β-arrestins in hypertension regarding HF. In the present review, we try to figure out the paradox between the roles of two isoforms of β-arrestin in the treatment of HF.

## 1. Introduction

Currently, heart failure (HF) is considered the most noteworthy global issue, regarded as the leading cause of annual deaths throughout the world despite notable development in the treatment of HF and responsible for a death every minute in the United States of America [1,2]. Bangladesh, a developing country, is amid threatening conditions due to an alarming increase in the rate of HF [3,4]. HF is a clinical syndrome characterized by plenty of factors that are detrimental to the cardiovascular system. From a physiological viewpoint, HF concerns the inadequate blood supply proportionate to the tissue’s metabolic needs, lowering the heart’s ability to pump or retrieve blood, leading to deficiency in the amount of oxygen necessary for the instigation of the normal functioning of vital organs and paving the way for dyspnea [5].

Like other clinical syndromes such as renal failure and hepatic disorder, HF has specific etiologies, though most of them are rarely covered. Yet, high blood pressure, coronary arterial disease, valvular heart diseases, cardiomyopathies, genetic cardiomyopathies, and primary idiopathic cardiomyopathy or other infiltrative disorders are thought to be major causes of HF [6]. Among them, increased blood pressure (i.e., hypertension) is perhaps the primary cause of HF [7]. The elevated volume and enlargement of the heart as a result of hypertension eventually lead to cardiac failure. The exact cause of hypertension is not discernable at all; however, different primary and secondary causes, such as genetic factors, fibromuscular dysplasia, primary aldosteronism, alcohol consumption, and the usage of over-the-counter (OTC) medications, are accountable for this clinical syndrome [8].

Researchers are focusing on several key aspects for the treatment of hypertension, which include discovering novel pathways, the formulation of groundbreaking drug delivery systems, and administration of multiple drugs for synergistic activities. Commencing with diuretics in the 1950s, the available medications so far for the treatment of hypertension include calcium channel blockers (CCBs), angiotensin receptor blockers (ARBs), angiotensin-converting enzyme (ACE) blockers, and beta-adrenergic blocking agents (β-blockers). However, these therapeutic agents need multiple administrations, consequently leading to medication intolerance. Recently, researchers have been working on several novel pathways to prevent or treat hypertension, and such pathways include Rho-kinase inhibition, antagonizing the mineralocorticoid receptor, and increasing the activity of enolase, a kidney-related peptide [9].

In contrast, frequent molecular targets used in modern drug therapy, such as the G-protein-coupled receptor (GPCR), are highly controlled by β-arrestins, which not only curb the activity of classic G-protein signaling but also commence definite β-arrestin signaling. The interaction between β-arrestin and G-protein signaling primarily regulates the cellular outcomes of GPCR-targeted drugs; therefore, β-arrestin signaling represents a thrilling place in research as a superior therapeutic target.

β-arrestin1 and β-arrestin2 are commonly recognized as inhibitors of GPCR signaling. As the GPCR becomes activated, the translocations of β-arrestins to the plasma membrane initiate binding with agonist-occupied receptors. This eventually unchains these specific G-protein receptors and assists their internalization, leading to desensitization. Activation of the G-protein results from binding agonists with receptors and leads to second-messenger signaling, which subsequently stimulates G-protein-coupled receptor kinases (GRKs) and induces phosphorylation [10]. Furthermore, the aforementioned receptors bind β-arrestin, which conformationally performs as an impediment for G-protein activity. Although β-arrestin interferes with G-protein signaling, it also makes a route for a cascade of signals. Two members of the arrestin family—β-arrestin1 and β-arrestin2 (also known as arrestin 2 and arrestin 3)— work as adaptor proteins for binding with GPCRs and initiate the formation of the complex [11]. However, recent works on the receptors, the β-2-adrenergic receptor (β2AR) and the muscarinic M1 receptor demonstrated the direct influence of β-arrestins on the second messenger’s degradation, employing phosphodiesterase (PDE) and diacylglycerol kinase, respectively, which implies a more multifaceted β-arrestin function in desensitization than was formerly understood [12,13].

This review aims to investigate the dual role of two forms of β-arrestin, namely β-arrestin1 and β-arrestin2, on blood pressure elevation and propose future research prospects for the prevention or treatment of HF through the regulation of hypertension.

## 2. Side Effects of Conventional Antihypertensive Drugs and an Insight on the Process of New Cardiac Drug Discovery

The conventional medications used so far for the treatment of hypertension mainly include calcium channel blockers (CCBs), angiotensin-converting enzyme (ACE) blockers, ARBs, and beta-adrenergic blocking agents (β-blockers). These agents might be threatening due to adverse effects, and several have a detrimental effect on the renal system in addition to being contraindicated in some special circumstances, including pregnancy and women of childbearing age [14]. A common adverse effect of antihypertensive therapy with vasodilators is vasodilatory edema. This phenomenon is related to several mechanisms, and most of them primarily include the stimulation of the renin–angiotensin–aldosterone system (RAAS), arteriolar dilatation, and fluid volume retention [15].

Lithell et al. proposed that the inept resistance of insulin and deterioration of lipoprotein metabolism both occur due to β-blockers and thiazide diuretics, and an epidemiological long-term follow-up study reported that hypertensive patients treated with β-blockers or diuretics gave rise to strong suspicion, which further enhanced the risk for developing diabetes [16].

Interestingly, the drug of choice in a diabetic patient with hypertension is ACE inhibitors to reduce diabetic nephropathy. However, all ACE inhibitors share plenty of potential adverse drug reactions, and these effects are substantially inherent in their mode of action. Specifically, patients with malignant hypertension might experience excessive hypotension. In addition, excessive hypotension is a possibility in patients with intense renin-dependent vasoconstriction, hyponatremia, and congestive heart failure (CHF). Moreover, patients using diuretics for their treatment as well as dehydrated patients, for various other reasons, may experience side effects [17]. Furthermore, hypotension may also rise from arteriosclerotic patients who have a vascular occlusive disease. After initiating ACE inhibitors, several other adverse reactions have been described, including coronary ischemia, angina, cerebral ischemia, and acute renal failure with transient oliguria.

Another potential adverse effect of ACE inhibition is hyperkalemia. Hyperkalemia is attributable to the secretion of aldosterone after angiotensin II (AngII) elimination, and a tiny elevation in plasma potassium ion (K^+^) levels can stimulate the secretion of aldosterone, which leads to new homeostasis and hyperkalemic levels unable to be reached in these circumstances. Nevertheless, the case is not the same for all patients. For instance, in elderly diabetic patients, patients with advanced renal insufficiency, or patients served with non-steroidal anti-inflammatory drugs (NSAIDs) or concomitant potassium-sparing diuretics, significantly acute hyperkalemia may develop [18]. Apart from this, angioedema [19,20] and a persistent dry cough along with wheezing have also been reported as a side effect of ACE inhibitors [21].

Blood pressure, along with electrolyte homeostasis, is regulated by the angiotensin II type 1 receptor (AT_1_R), which is the primary target of angiotensin receptor blockers (ARBs) for the mitigation of hypertension. In terms of an angiotensin receptor antagonist (ARA), the most prominent side effects associated with ACE inhibitors could be diminished, as ACE inhibitors’ side effects have been attributed to the activation of bradykinin, enkephalins, and other biologically active peptides. However, ARAs selectively block the AT_1_R and do not inhibit the catabolism of bradykinin. Although no other adverse effects are anticipated, extreme caution should be taken at the time of prescribing ARBs.

Cardiac AT_1_R has significant regulatory functions in the heart, especially in endothelial cells and cardiac fibroblasts as well as in cardiac myocytes, to a lesser extent [22,23,24,25]. However, the abundance of cardiac AT_1_R in adult human myocardium is remarkably lower compared with cardiac GPCR type β_1_AR (in the non-failing human heart, the density ratio of AT_1_R/β1AR is 1:15) [26].

The AngII peptide analog SII ([Sar1-Ile4-Ile8]-AngII), a β-arrestin-biased ligand which, upon binding to AT_1_R, elicits β-arrestin signaling without any Gq-protein signaling [27], paved the way for research on cardiac-biased AT_1_R signaling. In addition, β-arrestin recruitment to the AT_1_R mainly desensitizes Gq signaling and internalizes the receptor. Independent β-arrestin signaling of G-proteins is also induced by this [9,28,29]. This SII-stimulated signaling affirms that the conformations of the β-arrestin coupling receptor and the G-protein-activating receptor are distinct [8]. In contrast, β-arrestin’s effects in isolated adult mouse cardiomyocytes suggested that, in isolated cardiac myocytes, the contraction of β-arrestin signaling is induced via SII, indicating the cardiac role of β-arrestin-biased ligands [30]. Previously, it has been reported that the selective activation of AT_1_R-induced β-arrestin signaling in the heart and vasculature perhaps retains a better CVS effect [10,31,32,33]. In the case of human non-failing cardiomyocytes, the expression of β-arrestin1 is greater than that of β-arrestin2, so it might be possible that the “biased ligand” activation is mediated by β-arrestin1 instead of β-arrestin2 [34]. However, the AT_1_R- and β_1_AR-related previous works suggested the deleterious effect of classical G-protein signaling in HF, though in the case of β-arrestin-dependent, G-protein-independent signaling by these receptors, the roles were demonstrated to be cardioprotective [35,36]. As β_1_AR and AT_1_R chronic stimulation is detrimental in HF, inhibiting these receptors is considered the best therapy for heart disease. Although most of the ARBs and β-blockers curb the activity of both G-protein- and β-arrestin-mediated signaling, “biased ligands”, which specifically utilize β-arrestin signaling for their cardioprotective roles and simultaneously inhibit harmful G-protein signaling, can be recognized as novel receptor blockers. The AT_1_R is referred to as acting as a mechanosensor and functions as an active receptor. This mechanical stress induces β-arrestin-dependent signaling from the AT_1_R. ARB treatment reduces the effect of mechanically induced cardiac hypertrophy, and this indicates that ARBs display prevalently β-arrestin-biased inverse agonism [37]. It is expected that such a ligand(s) perhaps obstructs or alters detrimental cardiac remodeling due to excess circulatory catecholamines (CAs) and AngII in the heart. Previously, two such biased ligands have been documented. The AT_1_R ligand [Sar1, Ile4, Ile8]-ANG (SII) in particular activates β-arrestin-mediated signaling and inhibits G-protein-mediated signaling at the AT_1_R simultaneously in cardiac myocytes by increasing the beneficial effects of inotropy and positive lusitropy [38]. Additionally, recent studies reported that carvedilol improves mortality due to HF [39,40,41], which works by activating the β-arrestin signaling pathways poorly and blocking the G-protein signaling at the β_2_AR simultaneously [42]. The drug carvedilol is a weak β-arrestin-biased agonist which works by activating extracellular signal-regulated kinases (ERKs) via EGFR transactivation [42,43]. However, carvedilol is also a β2 and α1AR antagonist that may interfere with its β-arrestin-activating properties in the heart [44]. The addition of carvedilol to conventional therapy prolongs survival and improves the severity of heart failure. It has also been seen to reduce the risk of clinical deterioration, hospitalization, and serious adverse clinical events in patients who have symptoms at rest or upon minimal exertion [45].

A recent study on positive cardiac inotropy by carvedilol suggested that carvedilol-bound β1AR uniquely arouses β-arrestin2-dependent SERCA2a activity and the fractional shortening of cardiomyocytes in vitro [46]. Altogether, from the abovementioned information, it can be concluded that new therapeutic facilities in heart disease treatments can be pioneered by the discovery of β-arrestin-biased ARBs and β-blockers in the near future.

In addition, Gq/11-proteins and β-arrestins are crucial determinants for producing adrenal aldosterone and, importantly, both need to be deactivated for aldosterone production [47]. ARBs, acting as an AT_1_R inverse agonist, have not been included as β-arrestins activators thus far. Moreover, a recent review already stated that losartan (an ARB) is much more potent toward G-proteins than β-arrestins and act as a relatively G-protein biased agonist that cannot lower the production of aldosterone, which is mediated by β-arrestin [48]. However, two ARBs, namely candesartan and valsartan, displayed the highest agonism toward G-proteins and β-arrestins at the AT_1_R. Importantly, in vitro and in vivo findings depicted that the stated two ARBs were also effective in suppressing aldosterone, which means that both Gq-proteins and β-arrestins need to be suppressed to inhibit adrenal aldosterone synthesis [49,50,51]. Collectively, these abovementioned data suggest that the identification of β-arrestin-biased ARBs and β-blockers may pioneer new therapeutic opportunities regarding the treatment of heart diseases.

Considering the side effects of conventional antihypertensive drugs and the possible significant effect of β-arrestin ligand bias, we might conclude that these β-arrestin-biased ligands might have profound influences on the approach of researchers focusing on GPCR-targeted drug discovery.

## 3. Detrimental and Palliative Characteristics: A Paradox of the β-Arrestins Isoform

### 3.1. Functional Distinction between β-Arrestin1 and β-Arrestin2 in Cardiac Myocytes

β-arrestins are also termed non-visual arrestins, which are proteins expressed extensively in almost all tissues. The naming is due to the capability of arresting β2-adrenergic receptors (β2-ARs), as the role of β-arrestin includes the desensitization of β2-ARs.

β-arrestin1 shows cardiotoxic features. Genetic deletion of β-arrestin1 in the heart leads to many effective therapeutic consequences in HF, which include improvements in inotropic reserves along with cardiac βARs, enhanced severe adverse cardiac remodeling, and the elevated longevity of β-arrestin-1-knockout mice (β-arrestin1 KO) with post-myocardial infarction (MI) HF [52,53]. Additionally, it facilitates thrombosis in platelets [54], obstructs the potential electrolyte/water balance in the kidneys, and promotes aldosterone production in the adrenal gland as well as secretion induced by AngII. It also promotes flushing as a side effect, which is primarily caused by niacin [55].

However, β-arrestin2 was found to be cardioprotective, as it prevents cardiac inflammation and apoptosis in addition to tremendously weakening adverse remodeling post-MI [56]. Moreover, current findings indicate that β1AR-stimulated β-arrestin2 can enhance contractility, either in a direct mechanism or indirectly [57]. The direct effect was characterized by the interaction with sarco/endoplasmic reticulum Ca^2+^-ATPase (SERCA)-2a, stimulating small ubiquitin-related modifier (SUMO)ylation [38]. Reduced SERCA2a activity is a benchmark of heart failure, and SUMOylation is a critical post-translational modification, regulating the function of SERCA2a. Therefore, β-arrestin-2 directly stimulates SERCA2a activity, resulting in increased cardiac contractility [58].

Moreover, β-arrestin2 enhances cardiac function indirectly by departing from the β_1_AR-stimulated cyclic adenosine monophosphate (cAMP)-dependent pro-contractile signaling in vitro in cardiomyocytes and in vivo in post-MI HF mice [22].

**Figure 1:**
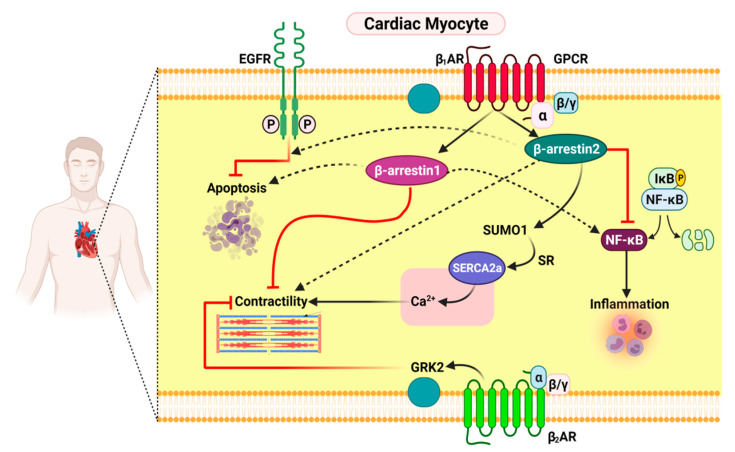
The functional distinction between β-arrestin1 and β-arrestin2 in cardiac myocytes. SR: sarcoplasmic reticulum; SUMO1: small ubiquitin-like modifier protein-1; EGFR: epidermal growth factor receptor; and AR: adrenergic receptor.

One of the mechanisms behind the anti-inflammatory effects of cardiac β-arrestin2 is nuclear factor-kappaB (NF-ĸB) inhibition in cardiac myocytes. This appears to be mediated only by β-arrestin2. β-arrestin-1 does not show this effect on the heart [59]. One of the principle mechanisms for the anti-apoptotic effects of cardiac β-arrestin2 is transactivation of the epidermal growth factor receptor (EGFR) by the cardiac β1AR [53], which is exclusive for β-arrestin2 because β-arrestin1 does not stimulate this; instead, it promotes cardiac apoptosis post-MI (Figure 1) [53,60].

Interestingly, as a result of non-failing human myocardium greatly under-expressing β-arrestin2, cardiac-specific pharmacological stimulation by β-arrestin2 or genetic transformation for HF therapy pioneered an excellent approach.

### 3.2. Functional Distinction between β-Arrestin1 and β-Arrestin2 in Cardiac AT_1_R Signaling and VSM

Despite their low abundance in adult human myocardium compared with cardiac GPCR-type β_1_AR (in the non-failing human heart, the density ratio of AT_1_R/β1AR is 1:15) [26], cardiac AT_1_R has significant regulatory functions in the heart, especially in endothelial cells, cardiac fibroblasts, and in cardiac myocytes to a lesser extent [22,23,24,25]. As we have already discussed in our previous section, β-arrestin recruitment to the AT_1_R mainly desensitizes Gq signaling and internalizes the receptor. Independent β-arrestin signaling of G proteins is also induced by this [9,28]. Other studies have shown that AT_1_R-elicited, β-arrestin-dependent signaling in cardiac myocytes results in cardiomyocyte proliferation without hypertrophy, which is Gq/11 protein signaling-dependent, may even lead to positive inotropy and lusitropy, depending on which GRK isoform is involved, but the case in which β-arrestin is engaged is also crucial [61,62]. However, on contractility in isolated Langendorff-perfused cardiac preparations, AT_1_R-activated β-arrestins have failed to show any effect [63].

Interestingly, GRK is potentially upregulated in the adrenal medulla during HF. Along with GRK2, β-arrestin1 is known to desensitize and downregulate adrenal α-2 adrenergic receptors (α2Ars), resulting in the chronic escalation of CA secretion, which elevates epinephrine and nor-epinephrine levels, thereby aggravating chronic HF [64,65,66,67].

Desensitization of adrenal α2Ars by β-arrestin1 plays a negative function in HF, and suppressing its activity on adrenal α2ARs (by preventing adrenal GRK2) can perhaps provide a platform for the design of novel therapeutic strategies for HF. In contrast, GRK2 and β-arrestin1 may reduce (whereas GRK6 and β-arrestin2 seem to promote) AT_1_R-induced contractility in primary murine adult cardiomyocytes [38].

**Figure 2:**
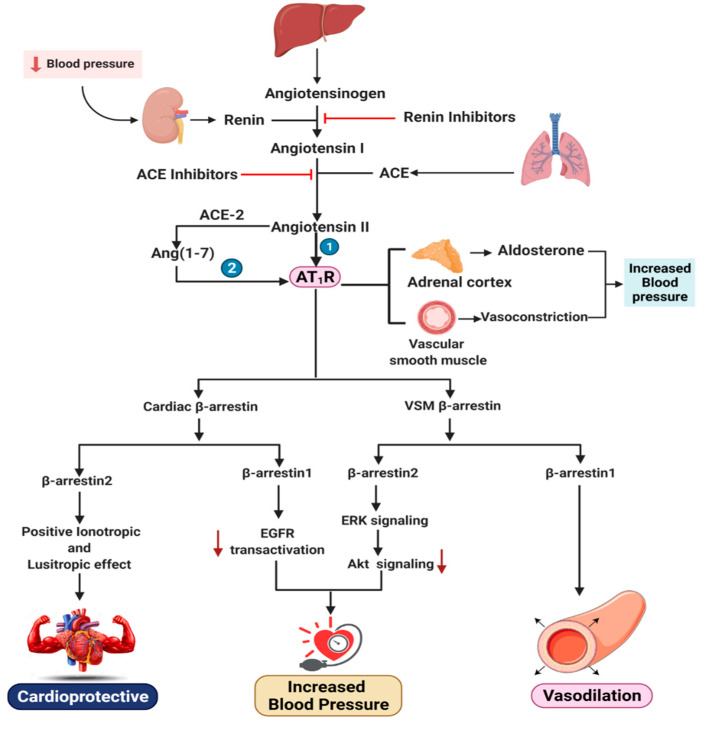
The renin–angiotensin–aldosterone system is a key regulator of blood pressure. Renin cleaves angiotensinogen into angiotensin I, which is further cleaved by ACE inhibitors to form angiotensin II. Here, two distinct pathways are possible. (1) Angiotensin II activates the AT_1_R and elicits vasoconstriction or aldosterone secretion, which eventually raises blood pressure. (2) Angiotensin II is cleaved by ACE2 or other carboxypeptidases to generate the heptapeptide Ang-(1–7), which is also responsible for β-arrestin1 and β-arrestin2 recruitment, giving both detrimental and salutary effects in terms of the cardiovascular system and VSM.

Inhibition of β-arrestin2 can be minimized in VSM to proliferate GPCR-stimulated VSM cells and activate ERK1/2; thus, β-arrestin2-dependent signaling plays a major role in injury response [68] (Figure 2). In particular, β-arrestin2 facilitates non-receptor Src protein kinase-dependent EGFR transactivation by the AT_1_R [69] to activate the small GTPase (monomeric G-protein) ADP-ribosylation factor (ARF)-642, as well as the upregulation of nuclear factor (NF)-kappa B-dependent cyclooxygenase (COX-2). As a result, β-arrestin2 stimulates VSM proliferation and hypertrophy, thus leading to the formation and growth of atheromas, vascular restenosis, and neointimal formation, while β-arrestin1 can counteract this detrimental activity [70,71,72,73,74].

VSM cells represent P2Y receptors, which stimulate vasoconstriction, hypertrophy, and hyperplasia. However, β-arrestin1 desensitizes the P2Y receptor and triggers vasodilatation in adverse conditions (hypertension or HF), which is in contrast to platelets, where P2Y receptor-activated β-arrestin1 induces thrombosis.

All of the aforementioned findings, when considered collectively, appear to develop a framework for cardiac β1AR signaling: (1) When β-arrestins (and especially β-arrestin2) regulate β1AR signaling, it is cardioprotective, but when G-proteins regulate β1AR signaling, it is cardiotoxic and induces apoptosis. (2) β1AR-induced β-arrestin1 is harmful to cardiac activity, as it reduces contractility, increases inflammation and apoptosis, and blocks the β-arrestin2-dependent signaling pathway, while β1AR-induced β-arrestin2 is effective on the heart due to EGFR transactivation and SERCA2a potentiation. Therefore, the β-arrestin1 restricting and β-arrestin2 boosting approaches suggest that β-arrestins can serve as a platform for the design of novel therapeutic strategies for HF.

Nonetheless, three factors must be taken into account when analyzing the clinical efficacy of cardiovascular β-arrestin targeting: (1) the type of tissue or cell, including cardiac fibroblast, cardiac myocyte, platelets, vascular endothelial cells, and VSM cells; (2) the β-arrestin isoform (β-arrestin1 or β-arrestin2); and (3) the type of GPCR. Moreover, parathyroid hormone receptor type 1, protease-activated receptors (PAR1, PAR2), and RTK toll-like receptor 4 are among the receptors where the isoforms of β-arrestin can perform inverse activities in signaling [75,76,77,78].

Future in vivo experiments are expected to explain the exact functions of the various β-arrestin isoforms in the cardiovascular system to treat cardiovascular diseases such as hypertension, HF, thromboembolism, atherosclerosis, and also cardiorenal disorders like nephrogenic diabetes insipidus.

## 4. Effects of β-Arrestin1 and β-Arrestin2 on the Renin–Angiotensin–Aldosterone System

The prominent hypertension mediator RAAS, which primarily maintains blood pressure, is a highly significant target for coronary disease. The RAAS cascade is caused by renin, an aspartic protease first isolated in 1898 from the crude rabbit adrenal cortex and synthesized in the juxtaglomerular cells in the afferent glomerular arteriole. Renin inhibition controls the RAAS mechanism, as renin cleaves the N-terminal of angiotensinogen, which is predominantly found in the liver but also in the kidney, heart, brain, placenta, ovary, adrenal gland, and adipose tissue. However, high angiotensinogen levels can be a concern for hypertension [79]. Renin cleaves angiotensinogen to form angiotensin-I (Ang I) or Ang-(1–10), an inert decapeptide that is hydrolyzed by angiotensin-converting enzyme (ACE) to produce the octapeptide angiotensin II (Ang II) or Ang-(1–8). Many enzymes like serine protease chymase have the same strength as ACE, where the activity of the chymase is not affected by ACE. Non-renin enzymes like cathepsin and tonin also interact with angiotensinogen directly to produce Ang II. Ang II is further affected by proteolytic cleavages that produce angiotensin III (Ang III) or Ang-(2–8) and angiotensin IV (Ang IV) or Ang (3–8).

ACE converts angiotensin I to angiotensin II (sequence: Asp-Arg-Val-Tyr-Ile-His-Pro-Phe), which is cleaved by ACE2 or several other carboxypeptidases and yields the heptapeptide Ang-(1–7) (sequence: Asp-Arg-Val-Tyr-Ile-His-Pro). Moreover, thimet oligopeptidase and several endopeptidases have been observed to have strong activity on Ang I for producing Ang-(1–7) [80,81,82,83].

Ang II binds to two types of Ang II receptors: Ang II type 1 (AT_1_) and Ang II type 2 (AT2), both of which are associated with the G-protein-coupled receptors (GPCRs) superfamily. Ang II activation has not been verified by any identified G-proteins or their downstream effectors. However, Ang II’s binds with the AT_1_ receptor (AT_1_R) and stimulates it to interact with the Gq-protein, which results in rising intracellular Ca^2+^, stimulating inositol trisphosphate and diacylglycerin and leading to the activation of several downstream effectors, phospholipases and intracellular protein kinase [84]. Primarily identified as a therapeutically functional peptide [85], angiotensin-(1–7) was later discovered to serve as an endogenous ligand and activator for the Mas receptor [86], which mainly consists of orphan GPCRs and was previously considered to be a proto-oncogen [87,88]. Activation of the Mas receptor increases the production of arachidonic acid but does not induce any of the recognized G-proteins. Furthermore, this activity induced by Ang II results in lessening the adverse cardiovascular effects, which allows vasodilation [89]. In terms of AT_1_R, Ang II’s last C-terminal amino acid residue (Phe8) affects the activation of G-protein signaling cascades, which exhibits the agonistic features of the ligand [90,91]. Ligands that trigger modifications at the Phe8 position, such as SII and TRV027, reduce G-protein signaling activation while fostering β-arrestin recruitment and signaling in the same period [10,92].

Ang-(1–7) is considered to bind with AT_1_R and serves as an agonist with unique activities, like β-arrestin-biased agonist properties. Though this activity does not activate the G-protein, it is highly stimulating to recruit and activate β-arrestin1 or β-arrestin2 and the phosphorylation of ERK1/2 [93,94,95].

It has been reported that among the variety of angiotensins, Ang II is the foremost effector of RAAS-related clinical conditions. AT_1_R mediates almost all the investigated roles of the RAAS. It is a classic GPCR, facilitating the phosphorylation of numerous proteins and regulating the entrance and translocation of calcium within the cell. AT_1_R, available in Ang II target organs, is responsible for hypertension, increased heart contractility, actions on the kidney, adrenal cortex, and sympathetic nervous system and the growth of Ang II.

As we have already discussed, unlike β-arrestin2, β-arrestin1 is somehow noxious to the myocardium, as it interferes with the function of extracellular signal-regulated kinase (ERK) signaling and epidermal growth factor receptor (EGFR) transactivation, which is crucial for cardiac sustainability as it impedes inflammation and apoptosis. However, the scenario is the opposite for AT_1_R located in vascular smooth muscle (VSM). β-arrestin2 is implicated with smooth muscle cell (SMC) proliferation in VSM through extracellular signal-regulated kinase and Akt activation, whereas β-arrestin1 is not. β-arrestin2 binds to the signaling protein ERK1/2, and ERK1/2 activation is dependent on its phosphorylation at Thr383, which actuates the Ang II-stimulated AT_1_R and controls the Akt pathway by forming a kinase–phosphatase scaffold [68,96]. In addition, Ang II corresponds to hypertension through ERK1/2 phosphorylation, but the effect is antagonized by an increase in the second messenger cellular adenosine 3,5-monophosphate (cAMP).

**Figure 3:**
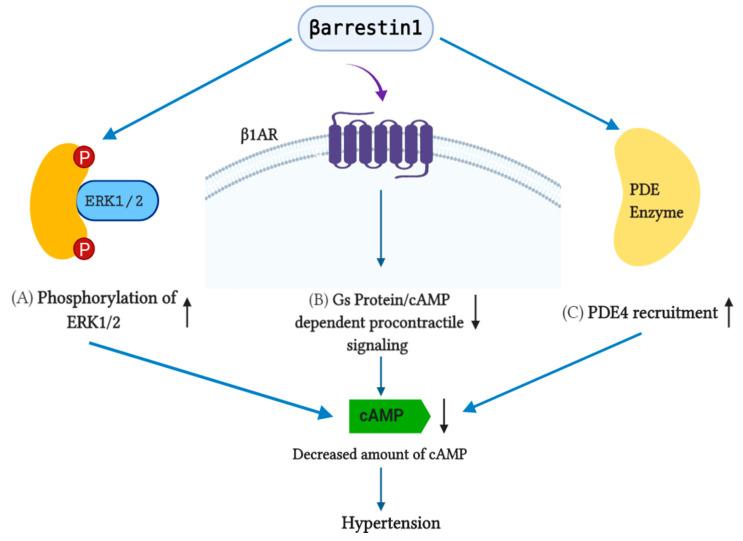
Effects of β-arrestin1 on the cellular cAMP level. (**A**) β-arrestin1 elevates the phosphorylation of ERK1/2, resulting in a decreased amount of cAMP. (**B**) β-arrestin 1 inhibits G_s_/protein/cAMP-dependent pro-contractile signaling, which eventually reduces the cellular cAMP level in β_1_AR. (**C**) β-arrestin1 involves PDE4 recruitment, ultimately causing a decreased cAMP level.

However, both β-arrestin1 and β-arrestin2 minimize cellular cAMP levels. β-arrestin2 is appointed as an active target in GPCR–cAMP–PKA signaling regulation. Its expression reduces cAMP signaling, and β-arrestin1 transfection ventilates the phosphorylation of ERK1/2, sustaining the activation of cAMP (Figure 3) [97].

The findings from Teixeira et al. depicted that Ang-(1–7) binds with AT_1_R but does not activate Gq, and it induces the recruitment and activation of β-arrestin1 and β-arrestin2. Moreover, in vivo studies have shown that Ang-(1–7) is effective for reducing the weight and thickness of the heart’s ventricular walls while also increasing the end-diastolic pressure, which helps to relieve heart hypertrophy [38].

The findings from Yang et al. have corroborated the phenomena, which demonstrated that [Pyr1]Apelin-13(1–12) is a stable ACE2 metabolite of [Pyr1]Apelin-13, an endogenous peptide involved in recruiting β-arrestin which is crucial for increasing ACE2 activity, resulting in cardioprotective consequences [98].

Aldosterone is a steroid hormone produced in the adrenal cortex’s zona glomerulosa. However, recent studies have found aldosterone in places other than the adrenal gland, such as the brain, heart, and blood vessels. It is already known that the development of hypertension is associated with aldosterone, which is the major hormone in the mineralocorticoid pathway that regulates blood pressure and volume. It is controlled by angiotensin II when other factors such as K^+^, Mg^2+^, serotonin, vasopressin, catecholamines, endothelin, and adrenocorticotropic hormone (ACTH) are involved in its secretion.

It is also associated with the development of hypertension and damage to targeted organs. Generally, aldosterone is considered to be synthesized in the adrenal glomerulosa and functions primarily with renal epithelial cells to facilitate Na^+^ and H_2_O retention and K^+^ and Mg^2+^ loss [99]. Aldosterone regulates the vascular tone by boosting the pressor response to catecholamines while lowering the vasodilatory response to acetylcholine or by stimulating the Ang II receptors. Moreover, higher aldosterone promotes vascular remodeling at the expense of arterial elasticity, causing collagen deposition in the blood vessels. On the other hand, chronic aldosterone administration in NaCl’s presence has been shown to induce perivascular and interstitial cardiac hypertrophy and fibrosis separate from the changes in blood pressure [100].

Interestingly, the mineralocorticoid receptor (MR) is considered to contribute to regulating aldosterone release from the RAAS, adding a new question in the role of aldosterone in hypertension studies by suggesting that antagonizing the MR might be conducive to reducing blood pressure. The adrenal gland is the main source of catecholamines (CAs), norepinephrine (NE), epinephrine (Epi), and aldosterone, which are causes of negative effects in remodeling in chronic heart failure (HF). Furthermore, in vivo studies on normal and post-myocardial infarction (MI) HF showed that βarrestine-1 is an important mediator for producing adrenocortical aldosterone [47,49] and adrenomedullary CA production [64,67]. However, a lack of β-arrestin1 from the heart improves β-AR-dependent cardiac function both physiologically and in post-MI HF at the same time. Though its deficiency in the adrenal gland decreases the circulating CA and aldosterone levels, it enhances the neurohormonal profile of the failing post-MI heart (Figure 3) [67].

Additionally, β-arrestin1, in combination with GRK2, is regarded as a tempting therapeutic target for HF, and blocking the activity of β-arrestin1 for aldosterone synthesis is perhaps much more efficient for the therapeutic threshold. Interestingly, the effect is not concerned with β-arrestin2, and the results from Selvaraj et al. have supported that the β-arrestin2 level was significantly decreased by the excess amount of aldosterone in the rat model [101].

Aldosterone controls the pathophysiological and cardiotoxic consequences of Ang II, and these levels rise in patients with chronic heart failure. For decades, it has been established that the AT_1_R is expressed endogenously in adrenocortical cells and stimulates adrenocortical zona glomerulosa cells for aldosterone production through Gq/11 protein-independent signaling, but with the β-arrestin1-dependent signaling pathway. However, AT_1_Rs can also send signals via β-arrestin1 or β-arrestin2, both of which regulate G-protein-independent signaling. Another AT_1_R-induced β-arrestin1-dependent signaling pathway and Gq/11 protein-independent signaling pathway, both of which operate in parallel and contribute to aldosterone synthesis and secretion, have been investigated in several studies. Moreover, ARB drugs known as AT_1_R antagonists inhibit both the aforementioned pathways in HF and suppress aldosterone in vitro and in vivo. AT_1_R antagonists such as irbesartan and losartan can block the Gq-mediated pathway, but their ability to inhibit β-arrestin1 and suppress aldosterone is minimal. However, another two potent ARBs, candesartan and valsartan, are more effective at restricting the β-arrestin1 pathway in vitro and in vivo. These two drugs have the potential to be effective aldosterone suppressors and treat other aldosterone-related conditions associated with higher aldosterone levels. This opens up the possibility of finding more chemical compounds that are similar to them [102].

## 5. Effects of β-Arrestin1 and β-Arrestin2 on Cardiac Beta-Adrenergic Receptors

β-adrenergic receptors (βARs) play a captivating role in the regulation of cardiovascular function, mainly regulating the heart rate and contractility. βARs belong to GPCRs, activated by catecholamines, and they stimulate the production of cAMP within the cell, thus regulating cardiac ion channel functioning, which leads to an increase in cardiac chronotropy and inotropy [103]. So far, three subtypes of βAR have been documented (β_1_AR, β_2_AR, and β_3_AR), of which β_1_AR is most abundant in cardiac myocytes at a ratio of approximately 4:1 with β_2_AR, whereas β_3_ARs are confined in adipose tissues [104]. The failing heart is activated adrenergically, and it has already been established that blocking βAR activities are appropriate for the treatment of hypertension and HF. Importantly, the binding affinity of β-arrestin2 with βARs is higher than β-arrestin1, which hinders the activation and normal function of β-arrestin1 [105]. Previous studies have delineated that the genetic deletion of cardiac β-arrestin1 results in potential therapeutic effects. However, β-arrestin2, which is less abundant in the heart than β-arrestin1, may possess cardioprotective effects [104]. Additionally, β-arrestin2 uniquely binds with several endoplasmic and sarcoplasmic reticulum-residing ATPases, interacting with the SUMOylation of SERCA2a and resulting in an uprise in the activity of the protein SERCA2a to exact positive inotropy, which is exactly the opposite of the function of β-arrestin1 [106]. In this review, we have already discussed the role of cAMP regarding hypertension. Following these functions, the results from McCrink et al. revealed that β-arrestin1 attenuates the production of cAMP related to β_1_AR in a murine model, whereas β-arrestin2 does not affect the β_1_AR-related cAMP related signaling. This strongly propounds that β-arrestin2 ameliorates cardiac survival, inflammation, apoptosis, and adverse remodeling post-MI, making β-arrestin2 pharmacological activation a positive inotropic strategy for the treatment of acute decompensated HF as well as hypertension and other cardiovascular conditions.

**Figure 4:**
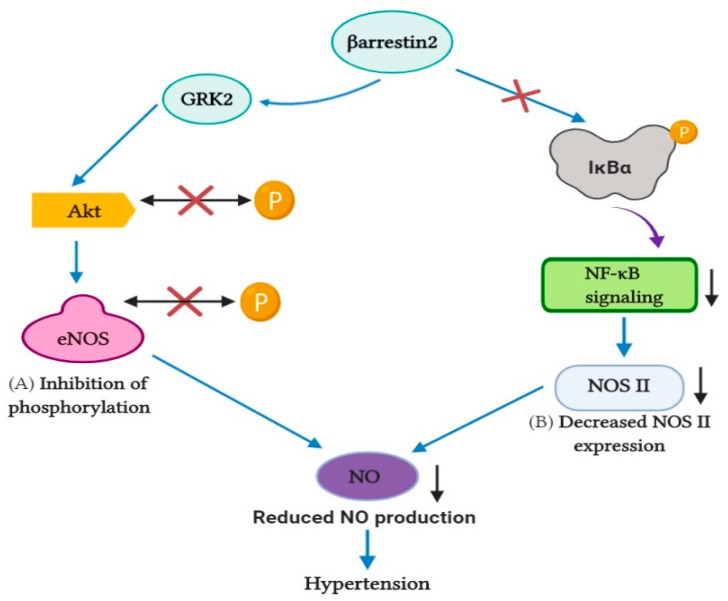
β-arrestin2 affects the production of NO. Along with GRK2, β-arrestin2 inhibits phosphorylation of the Akt/eNOS pathway, which decreases the amount of NO production. Furthermore, β-arrestin2 impedes NF-κB signaling by stabilizing IκBα, resulting in a decrease in NOS II expression and eventually decreasing the rate of NO production. Altogether, the result is increased blood pressure.

Desensitization of βARs occurs through myocardial GRKs, including GRK2, GRK3, and GRK5, which only recognize and phosphorylate agonist-induced receptors. Gros et al. described how increased GRK function is linked with elevated blood pressure in both the human and animal models [107]. GRK2, a sub-member of the GRK family, significantly leads to ventricular dysfunction, increased GRK2 activity contributes vastly to pathogenesis, and overexpression of GRK2 is positively regulated with blood pressure. Previous researchers have proposed the use of GRK2 as a potential medicinal approach in cardiovascular disease treatment, such as HF and hypertension [57,108]. Taguchi et al. found that the upregulation of GRK causes impairment or dysfunction in nitric oxide (NO) production [109]. GRK2 activity, along with β-arrestin2, triggers impairment in NO production through impeding the Akt/eNOS pathway by inhibiting the phosphorylation of Akt and eNOS, possibly causing hypertension. Previous studies have documented that GRK2 and β-arrestin2 compete to interact with receptors [110]. Kizaki et al. also presented that β-arrestin2 expression curbs the production of NO [111]. β-arrestin2 restrains nuclear factor kappa B (NF-κB) action via stabilizing cytoplasmic IκBα, lowering the chances of nitric oxide synthase (NOS) II expression, leading to a declivity in the production of NO, and possibly increasing risks for hypertension (Figure 4). Interestingly, β-arrestin1 has also delineated a hostile role in the production of NO, and previously, research has suggested that a β-arrestin1 shortage leads the way for extensive NO production [112].

## 6. TRV027, a Novel β-Arrestin-Biased Ligand: Progress and Failures

A peptide optimization campaign identified TRV027 (also known as TRV120027), a *β*-arrestin-biased ligand that is potent and selective [10] and both activates β-arrestins and inhibits G-proteins through the AT_1_R, was reported to produce moderate but significant acute positive inotropic and antiapoptotic effects in adult rodent hearts [32].

TRV027 reduces afterload, increasing cardiac performance while maintaining stroke volume, which is unlikely in the case of classical ARBs which, in normal animals, decrease cardiac contractility, cardiac output, and stroke volume [10]. TRV027 is a potent, balanced vasodilator that enhances cardiac output where the glomerular filtration rate was taken in while decreasing renal vascular resistance and increasing renal blood flow [31,113]. Studies have suggested that TRV027 could also reduce pulmonary capillary wedge pressure (a clinical correlate of dyspnea) as well as systemic and renal vascular resistance while increasing cardiac output [113].

Human studies of TRV027 have covered small, early phase studies in normal volunteers [114] as well as a phase IIa study in patients with HF and elevated filling pressures [115]. These early data suggested that TRV027 was tolerated well with a predictable pharmacokinetic profile. This ligand could also produce a rapidly reversible, dose-related decrease in the mean arterial pressure with no severe side effects.

Because of these previous favorable cardiovascular effects of TRV027, a clinical trial involving 620 adult patients with acute heart failure, named the Biased Ligand of the Angiotensin Receptor Study in Acute Heart Failure (BLAST-AHF), was directed [116]. Unfortunately, it was not able to meet either the primary or secondary endpoints in the phase IIb trial, suggesting that TRV027 is safe but not effective, at least in adults with acute heart failure.

There might be several reasons behind this. First, animal model findings do not always translate into humans the same way. Secondly, the compounds probably are not completely β-arrestin-biased (i.e., they may have had some weak, residual activity toward certain G-proteins). A recent review study by Lymperopoulos et al. on the functional diversity of the β-arrestins in the heart described an intriguing possibility behind the failure of the therapy, which is the significantly lower abundance of the cardioprotective β-arrestin2 compared with the cardiotoxic β-arrestin1 in human cardiomyocytes. They also suggested that these β-arrestin-biased compounds stimulated β-arrestin1 instead of β-arrestin2 in the patients’ hearts, which resulted in undesirable outcomes [117,118]. Finally, these biased ligands could stimulate the AT_1_R only in cardiac fibroblasts, which would remove any clinical benefit for ADHF patients. Summarizing all these findings, we could conclude that TRV027 may be a promising new drug for the treatment of ADHF, but further studies are warranted for a desirable outcome.

## 7. Effects of β-Arrestin1 and β-Arrestin2 on Renalase: A Novel Target for Hypertension

Renalase is a recently invented FAD-containing monoamine oxidase, a peptide related to the kidney. This enzyme is emitted in the blood and metabolizes the circulating catecholamines (CAs) [119]. The identification of renalase is considered a crucial step due to its relationship with the detailed understanding of cardiovascular physiology as well as renal function [9]. The relationship between renalase gene polymorphisms and disease development risks (e.g., hypertension, stroke, and coronary disease) with the left ventricular morphology held by SNPs was revealed in a number of recent studies.

A reduction effect by 28/20 mmHg of recombinant renalase (a single subcutaneous dose) on blood pressure which was similar to enalapril (5 mg/kg) was seen in a rat model of CKD (5/6 nephrectomy). The metabolization effect of renalase on circulating epinephrine and 1-3,4-dihydroxyphenylalanine can be concluded from this observation. In addition, the capability of renalase to reduce BP can also be directly correlated to its enzymatic activity [120]. Another rat model showed that a deficiency in renalase in the KO mouse was due to a significant rise in ischemic myocardial necrosis, although recombinant renalase administration entirely rescued this cardiac phenotype [121].

It has already been suggested that renalase lessens BP by two mechanisms: either by decreasing the heart rate and cardiac contractility or by forestalling the compensatory rises in peripheral vascular tone. Data obtained from the study by Li et al. have revealed that CAs regulate the production of renalase by stimulating enzymatic activity in the blood, increasing the secretion of renalase and activating renalase gene transcription, but this activated renalase breaks down the extracellular CAs [122]. Moreover, acute rises in the CA level in the blood significantly sustained the blood’s renalase activity.

**Figure 5:**
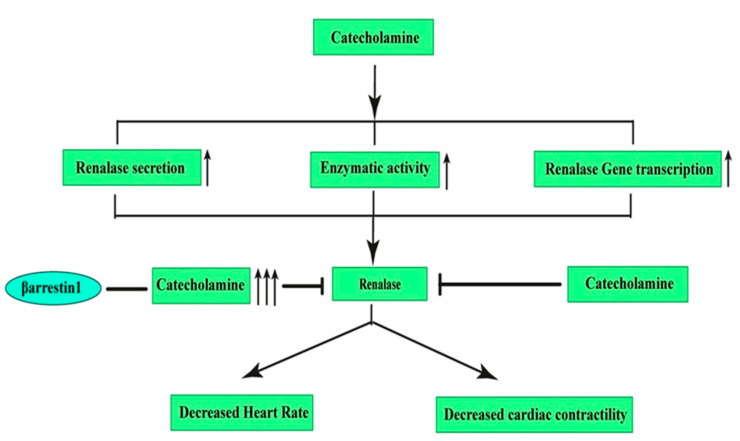
The renalase pathway. Catecholamines stimulate renalase synthesis through increasing renalase secretion from prorenalase, increased enzymatic activity, and enhanced gene transcription. Activated renalase then degrades catecholamines into their metabolites. Renalase decreases hypertension by decreasing the heart rate and cardiac contractility. However, β-arrestin1 expression causes an excessive amount of catecholamine synthesis, which further inhibits the beneficial action of renalase.

We have already discussed that β-arrestin1 in particular acts as a critical mediator of adrenocortical aldosterone production as well as of adrenomedullary CA production (serving in contact with adrenal GRK2) (Figure 5).

It is important to note that it has been reported that the β-arrestin1 knockout mice exhibited lower blood pressures consistent with the effect of circulating CAs, reducing the amount of circulating catecholamines [123]. Renalase decreases hypertension by decreasing the heart rate and cardiac contractility. However, β-arrestin1 expression causes an excessive amount of catecholamine synthesis, which further inhibits the beneficial action of renalase. This relationship could be of great use for paving the way to future drug discovery, though the effect of β-arrestin2 for this purpose has not been documented thus far and warrants further investigation.

## 8. Effects of β-Arrestin1 and β-Arrestin2 on Rho-Kinase

**Figure 6:**
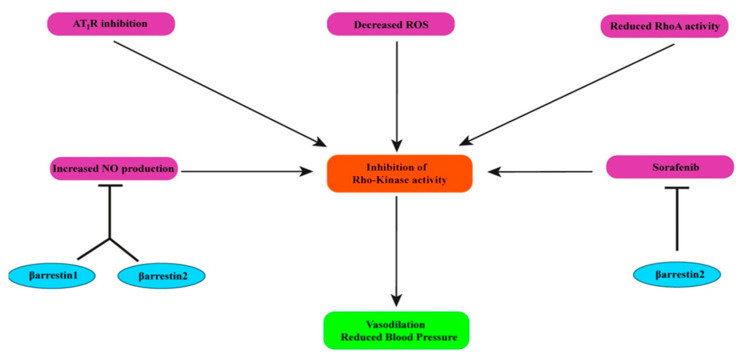
Inhibiting ROCK activity causes vasodilation, leading to blood pressure reduction. ROCK inhibition is mediated by declined RhoA activity, specific AT1R inhibition, a decreased amount of ROS, and increased NO production. However, both β-arrestin1 and β-arrestin2 are associated with decreasing the amount of NO. Moreover, β-arrestin2 impedes the activity of sorafenib, a ROCK inhibitor.

Rho-kinase (ROCK), a kinase which belongs to serine/threonine kinases, and the upregulation of ROCKs are associated with VSM contraction, which suggests the role of ROCK in hypertension. Blocking ROCK activity leads to blood pressure lowering effects. Additionally, several studies have documented that elevated RhoA activity is responsible for increased ROCK activity [124]. Previous studies have suggested that specific AT_1_R inhibition prevents ROCK and RhoA activity. As we have already discussed the controversial role of both β-arrestin isoforms on AT_1_R activity in the myocardium and VSM, determining the function of two β-arrestins may require further work for this issue. Aside from that, reactive oxygen species (ROS) formation also mediates a significant impact on the activation of RhoA and ROCKs, and oxidative stress is responsible for several types of hypertension in humans and animals [125,126]. Previous works have already suggested that ERK activation by β-arrestin2 causes the expression of ROCKs in vivo [127,128]. Moreover, sorafenib, a tyrosine kinase inhibitor that targets dysregulated ROCKs, is amenable to β-arrestin2, and the effects of sorafenib may be hindered by increased β-arrestin2 expression (Figure 6). This is presumably due to negative GPCR signaling and the boosted binding of β-arrestin2 with vasoconstrictor receptors in cirrhosis [129,130]. Additionally, evidence has revealed that NO inhibits RhoA/ROCK signaling. It has been demonstrated that the inactivation of RhoA/ROCK signaling is a part of NO-induced vasodilation. However, we have already shown that both isoforms of β-arrestin harm NO production. Furthermore, NF-κB is considered a potential biomarker for oxidative stress that is crucial for ROS accumulation [131]. In this review, we have already discussed that NF-κB action might be impeded by β-arrestin2. From this viewpoint, the role of β-arrestin2 is conducive for the inactivation of ROCKs. 

## 9. Conclusions

HF, considered a threat to modern society, is a systematic and complicated disease. The goal of HF treatment includes the identification of numerous pathways that are related to hypertension or other reasons. It is a challenging task which considers several mechanisms that regulate cardiac function differently. In this review, we are attempting to figure out the role of two β-arrestins isoforms: β-arrestin1 and β-arrestin2. Our investigation represents a paradox, showing both isoforms’ potential to treat HF related to hypertension, but it is β-arrestin2 which possesses more cardioprotective activities compared with its almost structurally similar analog β-arrestin1, although more detailed further investigations are warranted to conclude this. Hopefully, further in vivo analysis may ensure the precise roles of each β-arrestin isoform in every element of the cardiovascular system and validate at least some of the data in humans, too.

## Data Availability

Available data are presented in the manuscript.

## References

[1] Heron M.P. (2016). Deaths: Leading causes for 2013. Natl. Vital. Stat. Rep..

[2] Benjamin E.J., Blaha M.J., Chiuve S.E., Cushman M., Das S.R., Deo R., Floyd J., Fornage M., Gillespie C., Isasi C.R. (2017). Heart disease and stroke statistics-2017 update: A report from the American Heart Association. Circulation.

[3] Islam A.K.M.M., Mohibullah A.K.M., Paul T. (2016). Cardiovascular disease in Bangladesh: A review. Bangladesh Heart J..

[4] Khan A., Uddin R., Islam S.M.S. (2019). Clustering patterns of behavioural risk factors for cardiovascular diseases in Bangladeshi adolescents: A population-based study. Health Policy Technol..

[5] Savarese G., Lund L.H. (2017). Global Public Health Burden of Heart Failure. Card. Fail. Rev..

[6] Francis G.S., Tang W.H.W. (2019). Pathophysiology of congestive heart failure. Rev. Cardiovasc. Med..

[7] Messerli F.H., Rimoldi S.F., Bangalore S. (2017). The Transition from Hypertension to Heart Failure: Contemporary Update. JACC Heart Fail..

[8] Pucci M. (2019). Diagnosis and management of hypertension in primary care. Prescriber.

[9] Paulis L., Unger T. (2010). Novel therapeutic targets for hypertension. Nat. Rev. Cardiol..

[10] Violin J.D., DeWire S.M., Yamashita D., Rominger D.H., Nguyen L., Schiller K., Whalen E.J., Gowen M., Lark M.W. (2010). Selectively engaging β-arrestins at the angiotensin II type 1 receptor reduces blood pressure and increases cardiac performance. J. Pharmacol. Exp. Ther..

[11] Picard L.P., Schonegge A.M., Bouvier M. (2019). Structural Insight into G Protein-Coupled Receptor Signaling Efficacy and Bias between Gs and β-Arrestin. ACS Pharmacol. Transl. Sci..

[12] Nelson C.D., Perry S.J., Regier D.S., Prescott S.M., Topham M.K., Lefkowitz R.J. (2007). Targeting of diacylglycerol degradation to M1 muscarinic receptors by β-arrestins. Science.

[13] Perry S.J., Baillie G.S., Kohout T.A., McPhee I., Magiera M.M., Ang K.L., Miller W.E., McLean A.J., Conti M., Houslay M.D. (2002). Targeting of cyclic AMP degradation to beta 2-adrenergic receptors by beta-arrestins. Science.

[14] Girvin B. (2019). Hypertension clinic drug choices: Tips for pharmacist prescribers. Prescriber.

[15] Messerli F.H. (2002). Vasodilatory edema: A common side effect of antihypertensive therapy. Curr. Cardiol. Rep..

[16] Lithell H.O.L. (1991). Effect of antihypertensive drugs on insulin, glucose, and lipid metabolism. Diabetes Care.

[17] Frohlich E.D., Cooper R.A., Lewis E.J. (1984). Review of the Overall Experience of Captopril in Hypertension. Arch. Intern. Med..

[18] Ponce S.P., Jennings A.E., Madias N.E., Harrington J.T. (1985). Drug-Induced Hyperkalemia. Medicine.

[19] Davies R.O., Irvin J.D., Kramsch D.K., Walker J.F., Moncloa F. (1984). Enalapril worldwide experience. Am. J. Med..

[20] Jett G.K. (1984). Captopril-induced angioedema. Ann. Emerg. Med..

[21] Semple P.F., Herd G.W. (1986). Cough and Wheeze Caused by Inhibitors of Angiotensin-Converting Enzyme. N. Engl. J. Med..

[22] Maning J., Negussie S., Clark M.A., Lymperopoulos A. (2017). Biased agonism/antagonism at the AngII-AT1 receptor: Implications for adrenal aldosterone production and cardiovascular therapy. Pharmacol. Res..

[23] Lymperopoulos A. (2012). Beta-arrestin Biased Agonism/Antagonism at Cardiovascular Seven Transmembrane-spanning Receptors. Curr. Pharm. Des..

[24] Thomas W.G., Thekkumkara T.J., Baker K.M. (1996). Cardiac effects of AII: AT1A Receptor Signaling, Desensitization, and Internalization. Adv. Exp. Med. Biol..

[25] DeWire S.M., Ahn S., Lefkowitz R.J., Shenoy S.K. (2007). Beta-arrestins and cell signaling. Annu. Rev. Physiol..

[26] Kenakin T. (2005). New concepts in drug discovery: Collateral efficacy and permissive antagonism. Nat. Rev. Drug Discov..

[27] Fisher A., Heldman E., Gurwitz D., Haring R., Barak D., Meshulam H., Marciano D., Brandeis R., Pittel Z., Segal M. (1993). Selective signaling via unique M1 muscarinic agonists. Ann. N. Y. Acad. Sci..

[28] Violin J.D., Lefkowitz R.J. (2007). β-Arrestin-biased ligands at seven-transmembrane receptors. Trends Pharmacol. Sci..

[29] Violin J.D., Crombie A.L., Soergel D.G., Lark M.W. (2014). Biased ligands at G-protein-coupled receptors: Promise and progress. Trends Pharmacol. Sci..

[30] Fan H., Bitto A., Zingarelli B., Luttrell L.M., Borg K., Halushka P.V., Cook J.A. (2010). Beta-arrestin 2 negatively regulates sepsis-induced inflammation. Immunology.

[31] Boerrigter G., Whalen E.J., Lark M., Burnett J.C. (2010). Cardiorenal Actions of TRV120027, a Novel, β-Arrestin-Biased Ligand at the Angiotensin II Type I Receptor, in Healthy and Heart Failure Canines: A Novel Therapeutic Strategy for Acute Heart Failure. J. Card. Fail..

[32] Kim K.S., Abraham D., Williams B., Violin J.D., Mao L., Rockman H.A. (2012). β-arrestin-biased AT1R stimulation promotes cell survival during acute cardiac injury. Am. J. Physiol. Heart Circ. Physiol..

[33] Monasky M.M., Taglieri D.M., Henze M., Warren C.M., Utter M.S., Soergel D.G., Violin J.D., John Solaro R. (2013). The β-arrestin-biased ligand TRV120023 inhibits angiotensin II-induced cardiac hypertrophy while preserving enhanced myofilament response to calcium. Am. J. Physiol. Heart Circ. Physiol..

[34] McCrink K.A., Maning J., Vu A., Jafferjee M., Marrero C., Brill A., Bathgate-Siryk A., Dabul S., Koch W.J., Lymperopoulos A. (2017). Cardiac βarrestin2 Improves Contractility and Adverse Remodeling in Heart Failure, But Is Underexpressed in Humans. J. Am. Coll. Cardiol..

[35] Turu G., Balla A., Hunyady L. (2019). The Role of β-Arrestin Proteins in Organization of Signaling and Regulation of the AT1 Angiotensin Receptor. Front. Endocrinol..

[36] Zhai P., Yamamoto M., Galeotti J., Liu J., Masurekar M., Thaisz J., Irie K., Holle E., Yu X., Kupershmidt S. (2005). Cardiac-specific overexpression of AT1 receptor mutant lacking G αq/Gαi causes hypertrophy and bradycardia in transgenic mice. J. Clin. Investig..

[37] Wang J., Gareri C., Rockman H.A. (2018). G-protein-coupled receptors in heart disease. Circ. Res..

[38] Rockman H.A., Choi D.J., Akhter S.A., Jaber M., Giros B., Lefkowitz R.J., Caron M.G., Koch W.J. (1998). Control of myocardial contractile function by the level of beta-adrenergic receptor kinase 1 in gene-targeted mice. J. Biol. Chem..

[39] Hjalmarson A., Goldstein S., Fagerberg B., Wedel H., Waagstein F., Kjekshus J., Wikstrand J., El Allaf D., Vítovec J., Aldershvile J. (2000). Effects of controlled-release metoprolol on total mortality, hospitalizations, and well-being in patients with heart failure: The Metoprolol CR/XL Randomized Intervention Trial in congestive heart failure (MERIT-HF). MERIT-HF Study Group. JAMA.

[40] Investigators C., Commentary S. (1999). The Cardiac Insufficiency Bisoprolol Study II (CIBIS-II): A randomised trial. Lancet.

[41] Kveiborg B., Major-Petersen A., Christiansen B., Torp-Pedersen C. (2007). Carvedilol in the treatment of chronic heart failure: Lessons from the Carvedilol Or Metoprolol European Trial. Vasc Health Risk Manag..

[42] Wisler J.W. (2007). A unique mechanism of beta-blocker action: Carvedilol stimulates beta-arrestin signaling. Proc. Natl. Acad. Sci. USA.

[43] Kim I.M., Tilley D.G., Chen J., Salazar N.C., Whalen E.J., Violin J.D., Rockman H.A. (2008). β-Blockers alprenolol and carvedilol stimulate β-arrestin-mediated EGFR transactivation. Proc. Natl. Acad. Sci. USA.

[44] Carr R., Schilling J., Song J., Carter R.L., Du Y., Yoo S.M., Traynham C.J., Koch W.J., Cheung J.Y., Tilley D.G. (2016). Β-Arrestin-Biased Signaling Through the Β2-Adrenergic Receptor Promotes Cardiomyocyte Contraction. Proc. Natl. Acad. Sci. USA.

[45] Packer M., Fowler M.B., Roecker E.B., Coats A.J.S., Katus H.A., Krum H., Mohacsi P., Rouleau J.L., Tendera M., Staiger C. (2002). Effect of carvedilol on the morbidity of patients with severe chronic heart failure: Results of the carvedilol prospective randomized cumulative survival (COPERNICUS) study. Circulation.

[46] Lymperopoulos A., Desimine L., McCrink K.A., Maning J., Wertz S.L., Markan U., Pasupuleti S., Brill A., Parker B.M. (2018). Positive cardiac inotropy by carvedilol via unique beta-arrestin2-dependent SERCA2a stimulation. Eur. Heart J..

[47] Lymperopoulos A., Rengo G., Zincarelli C., Kim J., Soltys S., Koch W.J. (2009). An adrenal β-arrestin 1-mediated signaling pathway underlies angiotensin II-induced aldosterone production in vitro and in vivo. Proc. Natl. Acad. Sci. USA.

[48] Ferraino K.E., Cora N., Pollard C.M., Sizova A., Maning J., Lymperopoulos A. (2021). Adrenal angiotensin II type 1 receptor biased signaling: The case for “biased” inverse agonism for effective aldosterone suppression. Cell. Signal..

[49] Lymperopoulos A., Rengo G., Zincarelli C., Kim J., Koch W.J. (2011). Adrenal beta-arrestin 1 inhibition in vivo attenuates post-myocardial infarction progression to heart failure and adverse remodeling via reduction of circulating aldosterone levels. J. Am. Coll. Cardiol..

[50] Dabul S., Bathgate-Siryk A., Valero T.R., Jafferjee M., Sturchler E., Mcdonald P., Koch W.J., Lymperopoulos A. (2015). Suppression of adrenal ßarrestin1-dependent aldosterone production by ARBs: Head-to-head comparison. Sci. Rep..

[51] Lymperopoulos A., Sturchler E., Bathgate-Siryk A., Dabul S., Garcia D., Walklett K., Rengo G., McDonald P., Koch W.J. (2014). Different potencies of angiotensin receptor blockers at suppressing adrenal β-arrestin1-dependent post-myocardial infarction hyperaldosteronism. J. Am. Coll. Cardiol..

[52] Appear B. (1997). Knockout Mice; Stimulation, normal but demonstrate altered cardiac responses to beta-adrenergic Beta-arrestin1 knockout mice appear normal but demonstrate altered cardiac responses to beta-adrenergic stimulation. Circ. Res..

[53] Wang Y., Jin L., Song Y., Zhang M., Shan D., Liu Y., Fang M., Lv F., Xiao R.P., Zhang Y. (2017). β-arrestin 2 mediates cardiac ischemia-reperfusion injury via inhibiting GPCR-independent cell survival signalling. Cardiovasc. Res..

[54] Schaff M., Receveur N., Bourdon C., Ohlmann P., Lanza F., Gachet C., Mangin P.H. (2012). β-arrestin-1 participates in thrombosis and regulates integrin α IIbβ 3 signalling without affecting p2y receptors desensitisation and function. Thromb. Haemost..

[55] Walters R.W., Shukla A.K., Kovacs J.J., Violin J.D., DeWire S.M., Lam C.M., Chen J.R., Muehlbauer M.J., Whalen E.J., Lefkowitz R.J. (2009). β-Arrestin1 mediates nicotinic acid-induced flushing, but not its antilipolytic effect, in mice. J. Clin. Investig..

[56] Watari K., Nakaya M., Nishida M., Kim K.M., Kurose H. (2013). β-arrestin2 in Infiltrated Macrophages Inhibits Excessive Inflammation after Myocardial Infarction. PLoS ONE.

[57] Eckhart A.D., Ozaki T., Tevaearai H., Rockman H.A., Koch W.J. (2002). Vascular-targeted overexpression of G protein-coupled receptor kinase-2 in transgenic mice attenuates β-adrenergic receptor signaling and increases resting blood pressure. Mol. Pharmacol..

[58] Kho C., Lee A., Jeong D., Oh J.G., Chaanine A.H., Kizana E., Park W.J., Hajjar R.J. (2011). SUMO1-dependent modulation of SERCA2a in heart failure. Nature.

[59] Lee S.U., In H.J., Kwon M.S., Park B.O., Jo M., Kim M.O., Cho S., Lee S., Lee H.J., Kwak Y.S. (2013). β-Arrestin 2 mediates G protein-coupled receptor 43 signals to nuclear factor-κB. Biol. Pharm. Bull..

[60] Noma T., Lemaire A., Naga Prasad S.V., Barki-Harrington L., Tilley D.G., Chen J., Le Corvoisier P., Violin J.D., Wei H., Lefkowitz R.J. (2007). β-Arrestin-mediated β1-adrenergic receptor transactivation of the EGFR confers cardioprotection. J. Clin. Investig..

[61] Rajagopal K., Whalen E.J., Violin J.D., Stiber J.A., Rosenberg P.B., Premont R.T., Coffman T.M., Rockman H.A., Lefkowitz R.J. (2006). Beta-arrestin2-mediated inotropic effects of the angiotensin II type 1A receptor in isolated cardiac myocytes. Proc. Natl. Acad. Sci. USA.

[62] Zidar D.A., Violin J.D., Whalen E.J., Lefkowitz R.J. (2009). Selective engagement of G protein coupled receptor kinases (GRKs) encodes distinct functions of biased ligands. Proc. Natl. Acad. Sci. USA.

[63] Aplin M., Christensen G.L., Schneider M., Heydorn A., Gammeltoft S., Kjølbye A.L., Sheikh S.P., Hansen J.L. (2007). Differential extracellular signal-regulated kinases 1 and 2 activation by the angiotensin type 1 receptor supports distinct phenotypes of cardiac myocytes. Basic Clin. Pharmacol. Toxicol..

[64] Lymperopoulos A., Rengo G., Funakoshi H., Eckhart A.D., Koch W.J. (2007). Adrenal GRK2 upregulation mediates sympathetic overdrive in heart failure. Nat. Med..

[65] Lymperopoulos A., Rengo G., Gao E., Ebert S.N., Dorn G.W., Koch W.J. (2010). Reduction of sympathetic activity via adrenal-targeted GRK2 gene deletion attenuates heart failure progression and improves cardiac function after myocardial infarction. J. Biol. Chem..

[66] Lymperopoulos A., Rengo G., Koch W.J. (2007). Adrenal adrenoceptors in heart failure: Fine-tuning cardiac stimulation. Trends Mol. Med..

[67] Lymperopoulos A., Rengo G., Zincarelli C., Soltys S., Koch W.J. (2008). Modulation of adrenal catecholamine secretion by in vivo gene transfer and manipulation of G protein-coupled receptor kinase-2 activity. Mol. Ther..

[68] Kim J., Zhang L., Peppel K., Wu J.-H., Zidar D.A., Brian L., DeWire S.M., Exum S.T., Lefkowitz R.J., Freedman N.J. (2008). β-arrestins regulate atherosclerosis and neointimal hyperplasia by controlling smooth muscle cell proliferation and migration. Circ. Res..

[69] Charles R., Namkung Y., Cotton M., Laporte S.A., Claing A. (2016). β-Arrestin-mediated angiotensin II signaling controls the activation of ARF6 protein and endocytosis in migration of vascular smooth muscle cells. J. Biol. Chem..

[70] Morinelli T.A., Walker L.P., Velez J.C.Q., Ullian M.E. (2015). Clathrin-dependent internalization of the angiotensin II AT1A receptor links receptor internalization to COX-2 protein expression in rat aortic vascular smooth muscle cells. Eur. J. Pharmacol..

[71] Morinelli T.A., Lee M.H., Kendall R.T., Luttrell L.M., Walker L.P., Ullian M.E. (2013). Angiotensin II activates NF-κB through AT1A receptor recruitment of β-arrestin in cultured rat vascular smooth muscle cells. Am. J. Physiol. Cell Physiol..

[72] Ahn S., Kim J., Hara M.R., Ren X.R., Lefkowitz R.J. (2009). β-arrestin-2 mediates anti-apoptotic signaling through regulation of BAD phosphorylation. J. Biol. Chem..

[73] Kendall R.T., Lee M.-H., Pleasant D.L., Robinson K., Kuppuswamy D., McDermott P.J., Luttrell L.M. (2014). Arrestin-dependent angiotensin AT1 receptor signaling regulates Akt and mTor-mediated protein synthesis. J. Biol. Chem..

[74] Wilson P.C., Lee M.H., Appleton K.M., El-Shewy H.M., Morinelli T.A., Peterson Y.K., Luttrell L.M., Jaffa A.A. (2013). The arrestin-selective angiotensin AT1 receptor agonist [Sar1,Ile4,Ile8]-AngII negatively regulates bradykinin B2 receptor signaling via AT1-B2 receptor heterodimers. J. Biol. Chem..

[75] Kuo F.T., Lu T.L., Fu H.W. (2006). Opposing effects of β-arrestin1 and β-arrestin2 on activation and degradation of Src induced by protease-activated receptor 1. Cell. Signal..

[76] Kumar P., Lau C.S., Mathur M., Wang P., DeFea K.A. (2007). Differential effects of β-arrestins on the internalization, desensitization and ERK1/2 activation downstream of protease activated receptor-2. Am. J. Physiol. Cell Physiol..

[77] Sneddon W.B., Friedman P.A. (2007). Β-Arrestin-Dependent Parathyroid Hormone-Stimulated Extracellular Signal-Regulated Kinase Activation and Parathyroid Hormone Type 1 Receptor Internalization. Endocrinology.

[78] Fan H., Luttrell L.M., Tempel G.E., Senn J.J., Halushka P.V., Cook J.A. (2007). β-Arrestins 1 and 2 differentially regulate LPS-induced signaling and pro-inflammatory gene expression. Mol. Immunol..

[79] Atlas S.A. (2007). The renin-angiotensin aldosterone system: Pathophysiological role and pharmacologic inhibition. J. Manag. Care Pharm..

[80] Ferrario C.M., Chappell M.C. (1994). A new myocardial conversion of angiotensin I. Curr. Opin. Cardiol..

[81] Pereira M.G.A.G., Souza L.L., Becari C., Duarte D.A., Camacho F.R.B., Oliveira J.A.C., Gomes M.D., Oliveira E.B., Salgado M.C.O., Garcia-Cairasco N. (2013). Angiotensin II-independent angiotensin-(1-7) formation in rat hippocampus: Involvement of thimet oligopeptidase. Hypertension.

[82] Santos R.A., Brosnihan K.B., Jacobsen D.W., DiCorleto P.E., Ferrario C.M. (1992). Production of angiotensin-(1-7) by human vascular endothelium. Hypertension.

[83] Santos R.A. (2014). Angiotensin-(1-7). Hypertension.

[84] Porrello E.R., Delbridge L.M.D., Thomas W.G. (2009). The angiotensin II type 2 (AT2) receptor: An enigmatic seven transmembrane receptor. Front. Biosci..

[85] Ferrario C.M. (1998). Angiotension-(1-7) and antihypertensive mechanisms. J. Nephrol..

[86] Santos R.A.S., Simoes e Silva A.C., Maric C., Silva D.M.R., Machado R.P., De Buhr I., Heringer-Walther S., Pinheiro S.V.B., Lopes M.T., Bader M. (2003). Angiotensin-(1-7) is an endogenous ligand for the G protein-coupled receptor Mas. Proc. Natl. Acad. Sci. USA.

[87] Young D., Waitches G., Birchmeier C., Fasano O., Wigler M. (1986). Isolation and characterization of a new cellular oncogene encoding a protein with multiple potential transmembrane domains. Cell.

[88] Rabin M., Birnbaum D., Young D., Birchmeier C., Wigler M., Ruddle F.H. (1987). Human ros1 and mas1 oncogenes located in regions of chromosome 6 associated with tumor-specific rearrangements. Oncogene Res..

[89] Peña Silva R.A., Kung D.K., Mitchell I.J., Alenina N., Bader M., Santos R.A.S., Faraci F.M., Heistad D.D., Hasan D.M. (2014). Angiotensin 1-7 reduces mortality and rupture of intracranial aneurysms in mice. Hypertension.

[90] Noda K., Saad Y., Karnik S.S. (1995). Interaction of Phe8 of angiotensin II with Lys199 and His256 of AT1 receptor in agonist activation. J. Biol. Chem..

[91] Holloway A.C., Qian H., Pipolo L., Ziogas J., Miura S.I., Karnik S., Southwell B.R., Lew M.J., Thomas W.G. (2002). Side-chain substitutions within angiotensin II reveal different requirements for signaling, internalization, and phosphorylation of type 1A angiotensin receptors. Mol. Pharmacol..

[92] Wei H., Ahn S., Barnes W.G., Lefkowitz R.J. (2004). Stable interaction between β-arrestin 2 and angiotensin type 1A receptor is required for β-arrestin 2-mediated activation of extracellular signal-regulated kinases 1 and 2. J. Biol. Chem..

[93] Teixeira L.B., Parreiras E., Silva L.T., Bruder-Nascimento T., Duarte D.A., Simões S.C., Costa R.M., Rodríguez D.Y., Ferreira P.A.B., Silva C.A.A. (2017). Ang-(1-7) is an endogenous β-arrestin-biased agonist of the AT1 receptor with protective action in cardiac hypertrophy. Sci. Rep..

[94] Szakadáti G., Tóth A.D., Oláh I., Erdélyi L.S., Balla T., Várnai P., Hunyady L., Balla A. (2015). Investigation of the fate of type I angiotensin receptor after biased activation. Mol. Pharmacol..

[95] Tóth A.D., Turu G., Hunyady L., Balla A. (2018). Novel mechanisms of G-protein-coupled receptors functions: AT1 angiotensin receptor acts as a signaling hub and focal point of receptor cross-talk. Best Pract. Res. Clin. Endocrinol. Metab..

[96] Cassier E., Gallay N., Bourquard T., Claeysen S., Bockaert J., Crepieux P., Poupon A., Reiter E., Marin P., Vandermoere F. (2017). Phosphorylation of β-arrestin2 at Thr383 by MEK underlies β-arrestin-dependent activation of Erk1/2 by GPCRs. Elife.

[97] Wu H.X., Chen J.Y., Wang Q.T., Sun W.Y., Liu L.H., Zhang L.L., Wei W. (2012). Expression and function of β-arrestin 2 stimulated by IL-1β in human fibroblast-like synoviocytes and the effect of paeoniflorin. Int. Immunopharmacol..

[98] Yang P., Kuc R.E., Brame A.L., Dyson A., Singer M., Glen R.C., Cheriyan J., Wilkinson I.B., Davenport A.P., Maguire J.J. (2017). [Pyr1]apelin-13(1-12) is a biologically active ACE2 metabolite of the endogenous cardiovascular peptide [Pyr1]apelin-13. Front. Neurosci..

[99] Funder J.W. (1995). Mineralocorticoid receptors and hypertension. J. Steroid Biochem. Mol. Biol..

[100] Rocha R., Chander P.N., Zuckerman A., Stier C.T. (1999). Role of aldosterone in renal vascular injury in stroke-prone hypertensive rats. Hypertension.

[101] Selvaraj J., Sathish S., Mayilvanan C., Balasubramanian K. (2013). Excess aldosterone-induced changes in insulin signaling molecules and glucose oxidation in gastrocnemius muscle of adult male rat. Mol. Cell. Biochem..

[102] Lymperopoulos A., Aukszi B. (2017). Angiotensin receptor blocker drugs and inhibition of adrenal beta-arrestin-1-dependent aldosterone production: Implications for heart failure therapy. World J. Cardiol..

[103] Petrofski J.A., Koch W.J. (2003). The β-adrenergic receptor kinase in heart failure. J. Mol. Cell. Cardiol..

[104] Lymperopoulos A., Teplow D.B. (2018). Chapter Two—Arrestins in the Cardiovascular System: An Update. Progress in Molecular Biology and Translational Science.

[105] Zhabyeyev P., Zhang H., Oudit G.Y. (2017). Is β-Arrestin 2 a Magic Bullet for Heart Failure Treatment?. Hypertension.

[106] McCrink K.A., Maning J., Vu A., Jafferjee M., Marrero C., Brill A., Bathgate-Siryk A., Dabul S., Koch W.J., Lymperopoulos A. (2017). β-Arrestin2 Improves Post--Myocardial Infarction Heart Failure via Sarco (endo) plasmic Reticulum Ca2+-ATPase--Dependent Positive Inotropy in Cardiomyocytes. Hypertension.

[107] Gros R., Chorazyczewski J., Meek M.D., Benovic J.L., Ferguson S.S.G., Feldman R.D. (2000). G-Protein-Coupled Receptor Kinase Activity in Hypertension:Increased Vascular and Lymphocyte G-Protein Receptor Kinase-2 Protein Expression. Hypertension.

[108] Cannavo A., Komici K., Bencivenga L., D’amico M.L., Gambino G., Liccardo D., Ferrara N., Rengo G. (2018). GRK2 as a therapeutic target for heart failure. Expert Opin. Ther. Targets.

[109] Taguchi K., Kobayashi T., Takenouchi Y., Matsumoto T., Kamata K. (2011). Angiotensin II causes endothelial dysfunction via the GRK2/Akt/eNOS pathway in aortas from a murine type 2 diabetic model. Pharmacol. Res..

[110] Jorgensen R., Roed S.N., Heding A., Elling C.E. (2011). Beta-arrestin2 as a competitor for GRK2 interaction with the GLP-1 receptor upon receptor activation. Pharmacology.

[111] Kizaki T., Izawa T., Sakurai T., Haga S., Taniguchi N., Tajiri H., Watanabe K., Day N.K., Toba K., Ohno H. (2008). β2-Adrenergic receptor regulates Toll-like receptor-4-induced nuclear factor-κB activation through β-arrestin 2. Immunology.

[112] Tan S., Li L., Chen T., Chen X., Tao L., Lin X., Tao J., Huang X., Jiang J., Liu H. (2015). β-Arrestin-1 protects against endoplasmic reticulum stress/p53-upregulated modulator of apoptosis-mediated apoptosis via repressing p-p65/inducible nitric oxide synthase in portal hypertensive gastropathy. Free Radic. Biol. Med..

[113] Boerrigter G., Lark M.W., Whalen E.J., Soergel D.G., Violin J.D., Burnett J.C. (2011). Cardiorenal actions of TRV120027, a novel ß-arrestin-biased ligand at the angiotensin II type i receptor, in healthy and heart failure canines: A novel therapeutic strategy for acute heart failure. Circ. Heart Fail..

[114] Soergel D.G., Subach R.A., Cowan C.L., Violin J.D., Lark M.W. (2013). First clinical experience with TRV027: Pharmacokinetics and pharmacodynamics in healthy volunteers. J. Clin. Pharmacol..

[115] Soergel D., Subach R.A., James I.E., Cowan C.L., Gowen M., Lark M. (2013). Trvo27, a Beta-Arrestin Biased Ligand At the Angiotensin 2 Type 1 Receptor, Produces Rapid, Reversible Changes in Hemodynamics in Patients With Stable Systolic Heart Failure. J. Am. Coll. Cardiol..

[116] Pang P.S., Butler J., Collins S.P., Cotter G., Davison B.A., Ezekowitz J.A., Filippatos G., Levy P.D., Metra M., Ponikowski P. (2017). Biased ligand of the angiotensin II type 1 receptor in patients with acute heart failure: A randomized, double-blind, placebo-controlled, phase IIB, dose ranging trial (BLAST-AHF). Eur. Heart J..

[117] Lymperopoulos A., Wertz S.L., Pollard C.M., Desimine V.L., Maning J., McCrink K.A. (2019). Not all arrestins are created equal: Therapeutic implications of the functional diversity of the β arrestins in the heart. World J. Cardiol..

[118] Felker G.M., Butler J., Collins S.P., Cotter G., Davison B.A., Ezekowitz J.A., Filippatos G., Levy P.D., Metra M., Ponikowski P. (2015). heart failure therapeutics on thebasisofabiased ligand of theangiotensin-2 type 1 receptor. Rationale and design of the blast-ahf study (biased ligand of the angiotensin receptor study in acute heart failure). JACC Heart Fail..

[119] Xu J., Li G., Wang P., Velazquez H., Yao X., Li Y., Wu Y., Peixoto A., Crowley S., Desir G. (2005). V Renalase is a novel, soluble monoamine oxidase that regulates cardiac function and blood pressure. J. Clin. Investig..

[120] Desir G.V., Tang L., Wang P., Li G., Sampaio-Maia B., Quelhas-Santos J., Pestana M., Velazquez H. (2012). Renalase Lowers Ambulatory Blood Pressure by Metabolizing Circulating Adrenaline. J. Am. Heart Assoc..

[121] Wu Y., Xu J., Velazquez H., Wang P., Li G., Liu D., Sampaio-Maia B., Quelhas-Santos J., Russell K., Russell R. (2011). Renalase deficiency aggravates ischemic myocardial damage. Kidney Int..

[122] Li G., Xu J., Wang P., Velazquez H., Li Y., Wu Y., Desir G. (2008). V Catecholamines regulate the activity, secretion, and synthesis of renalase. Circulation.

[123] Bathgate-Siryk A., Dabul S., Pandya K., Walklett K., Rengo G., Cannavo A., De Lucia C., Liccardo D., Gao E., Leosco D. (2014). Negative impact of β-arrestin-1 on post-myocardial infarction heart failure via cardiac and adrenal-dependent neurohormonal mechanisms. Hypertension.

[124] Wirth A. (2010). Rho kinase and hypertension. Biochim. Biophys. Acta BBA Mol. Basis Dis..

[125] Lassègue B., Griendling K.K. (2004). Reactive oxygen species in hypertension; An update. Am. J. Hypertens..

[126] Touyz R.M., Schiffrin E.L. (2004). Reactive oxygen species in vascular biology: Implications in hypertension. Histochem. Cell Biol..

[127] De Godoy M.A.F., Patel C.A., Waldman S.A., Katsuki M., Regan R.F., Rattan S. (2007). H-ras inhibits RhoA/ROCK leading to a decrease in the basal tone in the internal anal sphincter. Gastroenterology.

[128] Hennenberg M., Trebicka J., Stark C., Kohistani A.Z., Heller J., Sauerbruch T. (2009). Sorafenib targets dysregulated Rho kinase expression and portal hypertension in rats with secondary biliary cirrhosis. Br. J. Pharmacol..

[129] Hennenberg M., Trebicka J., Sauerbruch T., Heller J. (2008). Mechanisms of extrahepatic vasodilation in portal hypertension. Gut.

[130] Hennenberg M., Trebicka J., Biecker E., Schepke M., Sauerbruch T., Heller J. (2007). Vascular dysfunction in human and rat cirrhosis: Role of receptor-desensitizing and calcium-sensitizing proteins. Hepatology.

[131] Bubici C., Papa S., Pham C.G., Zazzeroni F., Franzoso G. (2006). The NF-kB-mediated control of ROS and JNK signaling. Histol. Histopathol..

